# Analyzing the nonlinear association between length of hospital stay and post-stroke pneumonia risk a secondary analysis of the Henan Province stroke registry

**DOI:** 10.3389/fneur.2026.1711762

**Published:** 2026-02-04

**Authors:** Jian Li, Qiujing Li, Lichun Zhou

**Affiliations:** 1 Department of Neurology, Beijing Chao Yang Hospital, Capital University of Medical Sciences, Beijing, China; 2 Department of Public Laboratory, The Third People's Hospital of Kunming City, Infectious Disease Clinical Medical Center of Yunnan Province, Kunming, Yunnan, China

**Keywords:** acute ischemic stroke, length of hospital stay (LOS), multivariable logistic regression, nonlinear relationship, post-stroke pneumonia (SAP)

## Abstract

**Background and objective:**

Post-stroke pneumonia (SAP) is a common and serious complication in patients with acute ischemic stroke, associated with poor outcomes. Length of hospital stay (LOS) may influence SAP risk, but the dose–response relationship remains unclear. This study aimed to investigate the nonlinear association between LOS and SAP and identify potential inflection points.

**Methods:**

This secondary analysis of the Henan Province Stroke Registry included 926 acute ischemic stroke patients admitted to the First Affiliated Hospital of Zhengzhou University from January 2009 to December 2012. Multivariable logistic regression models were used to assess the association between LOS and SAP. A two-piecewise linear model was applied to detect threshold effects, with multiple imputation for missing data.

**Results:**

The overall SAP incidence was 20.4%. After adjusting for age, sex, comorbidities, and NIHSS score, each additional day of LOS increased SAP risk by 8.3% (adjusted *OR* = 1.083; 95% *CI*: 1.057–1.110). Nonlinear analysis revealed a significant two-phase relationship with an inflection point at 17 days: *OR* = 1.182 (95% *CI*: 1.098–1.273) for LOS < 17 days, and *OR* = 1.049 (95% *CI*: 1.015–1.084) for ≥17 days (*P* for log-likelihood ratio test = 0.010).

**Conclusion:**

LOS is positively associated with SAP in a nonlinear manner, with a steeper risk increase during the first 17 days of hospitalization. These findings suggest intensified respiratory monitoring early in admission and optimized hospitalization strategies to reduce infection risk.

## Introduction

Acute ischemic stroke accounts for approximately 87% of all strokes and remains a leading global cause of mortality and disability (1). The Global Burden of Disease Study 2021 reported 14.3 million new stroke cases annually, with stroke contributing to about 11% of worldwide deaths—second only to ischemic heart disease (2). In China, the burden is rising due to aging and increasing prevalence of key risk factors such as hypertension, diabetes, and atrial fibrillation (3). Identifying modifiable process-of-care variables to improve outcomes is therefore of major public health importance.

Length of hospital stay (LOS) may influence recovery through multiple pathways (4). Early discharge with prompt rehabilitation can enhance neuroplasticity, as shown by EEG evidence of synaptic reorganization following early motor interventions (5); Conversely, prolonged LOS often signals complications—such as stroke-associated pneumonia (SAP), deep vein thrombosis, or systemic inflammation—that are linked to worse prognosis (6). Extended stays may also contribute to cognitive decline through social isolation and sensory deprivation (7).

Although LOS is widely used as a measure of healthcare efficiency, its direct association with clinical outcomes as an independent predictor remains unclear (8). While specialized stroke units have been shown to reduce LOS and mortality—suggesting LOS may reflect care quality—prior studies often treat LOS as a secondary measure and inadequately adjust for critical confounders like NIHSS score, reperfusion therapy, and complications. Consequently, few analyses have rigorously evaluated LOS as the primary exposure of interest in relation to specific stroke-related outcomes like stroke-associated pneumonia (9).

We conducted a secondary analysis of an existing cohort of 926 patients with acute ischemic stroke admitted to the First Affiliated Hospital of Zhengzhou University between January 2009 and December 2012 to examine the association between length of hospital stay (LOS) and the risk of in-hospital stroke-associated pneumonia (SAP)—defined as pneumonia diagnosed during the index admission based on CDC-aligned clinical, laboratory, and imaging criteria. Multivariable regression models were used to account for a range of baseline clinical and stroke-related confounders. Given that LOS may reflect modifiable aspects of inpatient care, this analysis aims to contribute to the ongoing discussion on how hospital-level processes influence outcomes in acute stroke management.

## Method

### Study population

This study is a secondary analysis of data from the Henan Province Stroke Registry, a prospective cohort originally designed to externally validate the A^2^DS^2^ score for predicting SAP in a Chinese population (10). The registry enrolled consecutive adult patients (aged ≥18 years) hospitalized for acute ischemic stroke or transient ischemic attack at the First Affiliated Hospital of Zhengzhou University (a tertiary center in central China) between January 2009 and December 2012, with stroke onset to admission ≤7 days.

Acute ischemic stroke was diagnosed by neurologists based on clinical evaluation and confirmed by brain imaging (CT or MRI). SAP was defined as hospital-acquired pneumonia meeting Centers for Disease Control and Prevention (CDC) criteria, excluding pre-admission pneumonia. Data were prospectively collected using standardized case report forms by trained staff blinded to study hypotheses.

**Figure 1:**
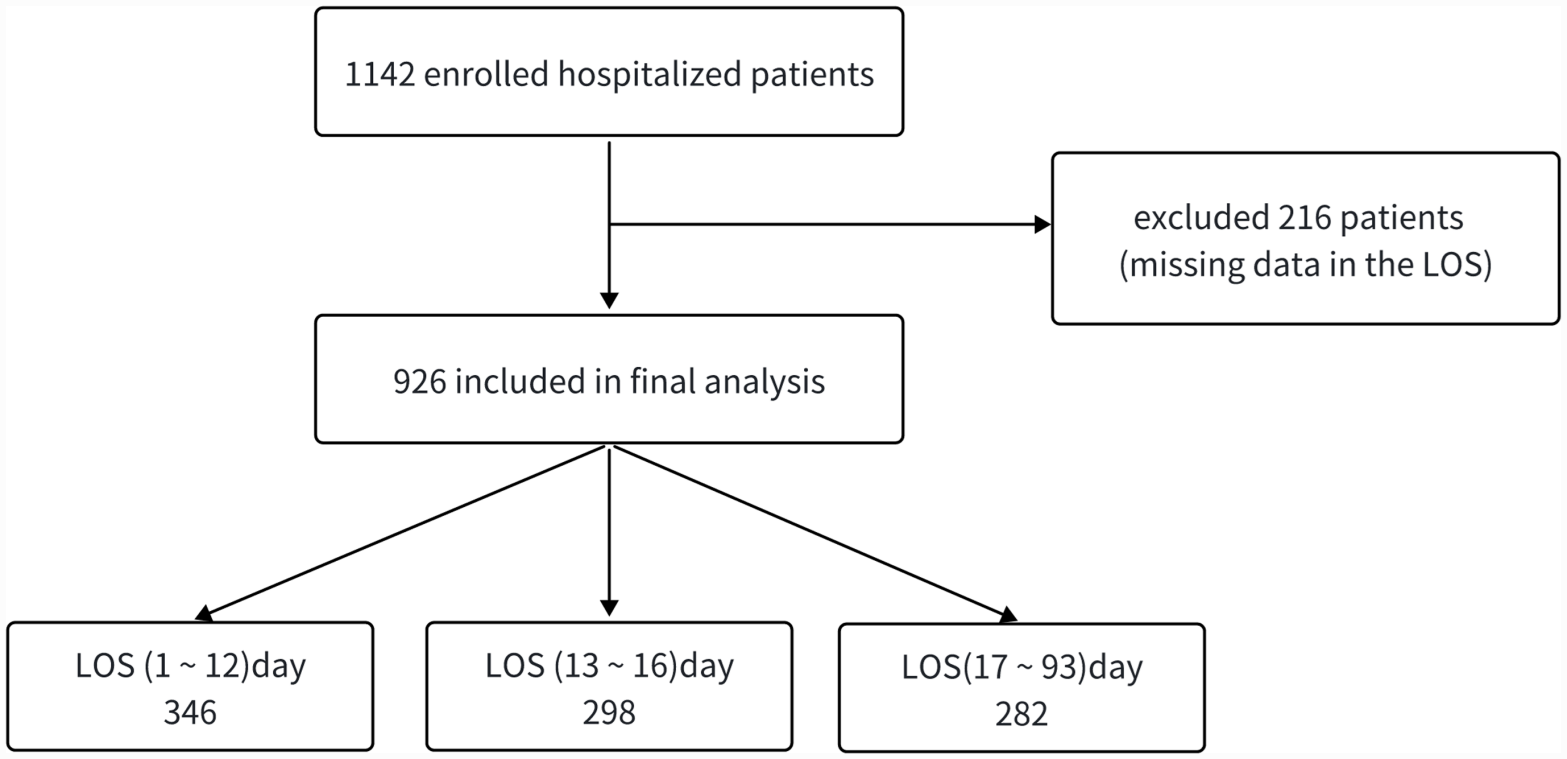
Flowchart of study population. The flowchart was used to illustrate how the study population was selected.

After exclusions (missing LOS or SAP data, hemorrhagic stroke, or incomplete covariate records), 926 patients were included in the final analysis (Figure 1). Due to the original registry design focused on routinely available variables, chronic obstructive pulmonary disease, congestive heart failure, pre-stroke modified Rankin Scale (mRS), and Glasgow Coma Scale (GCS) were not systematically recorded and were unavailable for the current analysis.

### Variables

The primary exposure variable was LOS, defined as the number of days from hospital admission to discharge following acute ischemic stroke. LOS was recorded as a continuous variable in days, extracted directly from the hospital’s electronic health record system on the day of discharge. To evaluate potential nonlinear effects and clinical thresholds, LOS was also categorized into three groups—low (1–12 days), medium (13–16 days), and high (≥17 days)—based on distribution patterns and inflection points identified in sensitivity analyses.

The primary outcome was SAP, defined as new-onset, hospital-acquired pneumonia occurring during the index hospitalization for acute ischemic stroke. SAP was diagnosed by the treating physician based on the Centers for Disease Control and Prevention (CDC) criteria for hospital-acquired pneumonia, including clinical and laboratory evidence of respiratory tract infection (e.g., fever, cough, new purulent sputum, auscultatory crackles, or positive sputum culture) supported by typical findings on chest X-ray or CT. Pneumonia present prior to stroke onset or at admission was explicitly excluded. The exact timing of SAP diagnosis (e.g., days post-stroke) was not recorded in the dataset.

Covariates were selected based on established clinical relevance and prior evidence linking them to either stroke severity or infection risk. These included age (categorized as <65 vs. ≥65 years), sex, hypertension, diabetes mellitus, coronary heart disease, history of prior stroke or transient ischemic attack (TIA), smoking status, presence of dysphagia assessed within 24 h of admission via bedside water swallow test, and the National Institutes of Health Stroke Scale (NIHSS) score. The NIHSS score was recorded at hospital admission, before any acute intervention, and served as a measure of baseline stroke severity. NIHSS score was categorized into three clinically meaningful levels—mild (0–4), moderate (5–12), and severe (≥12)—following conventional thresholds widely used in stroke research and consistent with prior analyses in Chinese stroke cohorts (11), including the original Henan Province Stroke Registry. All covariates were extracted from structured fields in the electronic medical record or derived from physician documentation. Missing data were minimal across variables (<5%), and multiple imputation by chained equations (MICE) was applied to generate five complete datasets, preserving statistical power and reducing potential bias.

#### Statistical analysis

Analyses were performed using Stata 15.0 (StataCorp, TX, USA) or R (version 4.2.0). *p* values <0.05 (two-sided) were considered statistically significant. The association between length of hospital stay (LOS, continuous, per 1-day increase) and stroke-associated pneumonia (SAP) was evaluated using multivariable logistic regression. The primary model was adjusted for age (continuous), sex, hypertension, diabetes mellitus, dyslipidemia, atrial fibrillation, coronary heart disease, history of stroke/TIA, current smoking, NIHSS score on admission (continuous), dysphagia, and OCSP stroke subtype (TACI, PACI, POCI, LACI). Multicollinearity was assessed by variance inflation factors (VIF < 5). Missing data were handled by multiple imputation using chained equations (20 imputations).

Nonlinearity was examined with a two-piecewise logistic regression model; the inflection point was selected via likelihood ratio test and grid search. Exploratory subgroup analyses tested effect modification by adding individual LOS × covariate interaction terms (sex, history of stroke/TIA, coronary heart disease, hypertension, diabetes, atrial fibrillation, dysphagia, NIHSS categories) to the fully adjusted primary model one at a time. *p*-values for interaction were derived from likelihood ratio tests. Interaction terms were not simultaneously included in the primary model due to the exploratory nature of these analyses and to prevent overfitting (189 SAP events).

#### Ethics statement

The original prospective cohort study was approved by the Institutional Review Board of the First Affiliated Hospital of Zhengzhou University and conducted in accordance with the Declaration of Helsinki. Written informed consent was obtained from all participants or their legally authorized representatives prior to enrollment in the primary study (10).

The present study is a secondary analysis of fully anonymized data from this previously published registry. No additional ethical approval or participant consent was required, as confirmed by the local ethics committee. All personal identifiers were removed prior to analysis, and data access was restricted to authorized personnel to ensure privacy and confidentiality.

## Results

This study enrolled 926 patients with acute ischemic stroke to examine the association between LOS and SAP. Patients were grouped by LOS: low (1–12 days, *n* = 346), medium (13–16 days, *n* = 298), and high (≥17 days, *n* = 282). Baseline characteristics showed a significant increase in SAP incidence with longer LOS (low: 8.7%, medium: 15.1%, high: 40.4%; *p* < 0.001). Proportions of dysphagia, diabetes, higher NIHSS scores, and OCSP classification as POCI also increased with LOS (all *p* < 0.05). Age, sex, and hypertension were balanced across groups (*p* > 0.05) (Table 1).

**Table 1:** Baseline characteristics of study participants by LOS.

Characteristic	Overall	LOS			*p* value
		Low (1.00 ~ 12.00)	Medium (13.00 ~ 16.00)	High (17.00 ~ 93.00)	
*N*	926	346	298	282	
SAP, *n* (%)	189 (20.4%)	72 (8.7%)	17 (15.1%)	100 (40.4%)	
Age					0.626
<65	562 (60.7%)	209 (60.4%)	187 (62.8%)	166 (58.9%)	
>65	364 (39.3%)	137 (39.6%)	111 (37.2%)	116 (41.1%)	
Sex					0.378
Male	340 (36.7%)	126 (36.4%)	118 (39.6%)	96 (34.0%)	
Female	586 (63.3%)	220 (63.6%)	180 (60.4%)	186 (66.0%)	
Dysphagia					<0.001
No	740 (79.9%)	299 (86.4%)	259 (86.9%)	182 (64.5%)	
Yes	186 (20.1%)	47 (13.6%)	39 (13.1%)	100 (35.5%)	
Hypertension					0.304
No	385 (41.6%)	155 (44.8%)	119 (39.9%)	111 (39.4%)	
Yes	541 (58.4%)	191 (55.2%)	179 (60.1%)	171 (60.6%)	
DM					0.001
No	650 (70.2%)	267 (77.2%)	204 (68.5%)	179 (63.5%)	
Yes	276 (29.8%)	79 (22.8%)	94 (31.5%)	103 (36.5%)	
History of Stroke/TIA					0.624
No	664 (71.7%)	248 (71.7%)	219 (73.5%)	197 (69.9%)	
Yes	262 (28.3%)	98 (28.3%)	79 (26.5%)	85 (30.1%)	
CHD					0.830
No	840 (90.7%)	316 (91.3%)	268 (89.9%)	256 (90.8%)	
Yes	86 (9.3%)	30 (8.7%)	30 (10.1%)	26 (9.2%)	
Smoking					0.342
Non-current	666 (71.9%)	258 (74.6%)	207 (69.5%)	201 (71.3%)	
Yes	260 (28.1%)	88 (25.4%)	91 (30.5%)	81 (28.7%)	
OCSP types					<0.001
LACI	191 (20.6%)	57 (16.5%)	80 (26.8%)	54 (19.1%)	
PACI	338 (36.5%)	134 (38.7%)	103 (34.6%)	101 (35.8%)	
TACI	29 (3.1%)	3 (0.9%)	6 (2.0%)	20 (7.1%)	
POCI	221 (23.9%)	91 (26.3%)	64 (21.5%)	66 (23.4%)	
NIHSS score					<0.001
Mild	603 (65.1%)	260 (75.1%)	212 (71.1%)	131 (46.5%)	
Moderate	227 (24.5%)	66 (19.1%)	62 (20.8%)	99 (35.1%)	
Severe	96 (10.4%)	20 (5.8%)	24 (8.1%)	52 (18.4%)	

Values are *n* (%) for categorical variables and mean ± SD or median (IQR) for continuous variables.

DM, Diabetes Mellitus; SD, standard deviation; IQR, interquartile range, LOS, length of stay; SAP, stroke-associated pneumonia.

*p* values were derived from *χ*^2^ tests for categorical variables, one-way ANOVA for normally distributed continuous variables, and Kruskal-Wallis H tests for non-normally distributed continuous variables.

Univariate analysis showed that each additional day of hospitalization was associated with a 10% increase in SAP risk (*OR* = 1.10, 95% *CI*: 1.081–1.128, *p* < 0.001) (Table 2). After adjusting for age, sex, comorbidities, and other clinically relevant factors in multivariable logistic regression, LOS remained significantly associated with SAP (adjusted *OR* = 1.083, 95% *CI*: 1.057–1.110, *p* < 0.001), indicating that LOS is an independent predictor of SAP (Table 3).

**Table 2:** Univariate analysis.

Variable	Estimate	95% CI	*p* value
LOS	1.10	(1.081, 1.128)	<0.001
Age			
< 65 years	1.0		
≥ 65 years	1.96	(1.419, 2.705)	<0.001
Sex			
Male	1.0		
Female	0.93	(0.668, 1.291)	0.660
Dysphagia			
No	1.0		
Yes	23.5	(15.720, 35.247)	<0.001
Hypertension			
No	1.0		
Yes	1.02	(0.734, 1.405)	0.924
DM			
No	1.0		
Yes	1.61	(1.150, 2.248)	0.005
History of stroke/TIA			
No	1.0		
Yes	1.67	(1.178, 2.361)	0.004
CHD			
No	1.0		
Yes	1.04	(0.600, 1.788)	0.900
Smoking			
Non-current	1.0		
Yes	0.90	(0.629, 1.295)	0.578
OCSP types			
LACI	1.0		
PACI	2.12	(1.265, 3.539)	0.004
TACI	6.24	(2.651, 14.692)	<0.001
POCI	2.61	(1.523, 4.464)	0.001
NIHSS score			
0–4	1.0		
5–12	3.94	(2.667, 5.830)	<0.001
>12	16.8	(10.227, 27.641)	<0.001

DM, Diabetes Mellitus; OR, odds ratio; HR, hazard ratio; CI, confidence interval; CHD, coronary heart disease; OCSP, Oxford Community Stroke Project; NIHSS, National Institutes of Health Stroke Scale; LOS, length of stay; SAP, stroke-associated pneumonia.

All models were adjusted for none.

Statistical significance was set at *p* < 0.05 (two-sided).

**Table 3:** Multivariable regression analysis of LOS associated with SAP.

Characteristic	Unadjusted OR(95% CI)	*p* value	Adjusted OR (95% CI)	*p* value
LOS^a^	1.104 (1.081, 1.128)	<0.0001	1.083 (1.057, 1.109)	<0.0001

OR, odds ratio; HR, hazard ratio; CI, confidence interval, OS, length of stay; SAP, stroke-associated pneumonia.

Adjusted for age (continuous), sex (male vs. female), hypertension (yes/no), diabetes mellitus (yes/no), dyslipidemia (yes/no), atrial fibrillation (yes/no), coronary heart disease (yes/no), history of stroke/TIA (yes/no), current smoking (yes/no), NIHSS score on admission (continuous), dysphagia (yes/no), and stroke subtype according to OCSP classification (TACI, PACI, POCI, LACI). All variables had variance inflation factors (VIF).^a^indicates *p* < 0.05.

**Figure 2:**
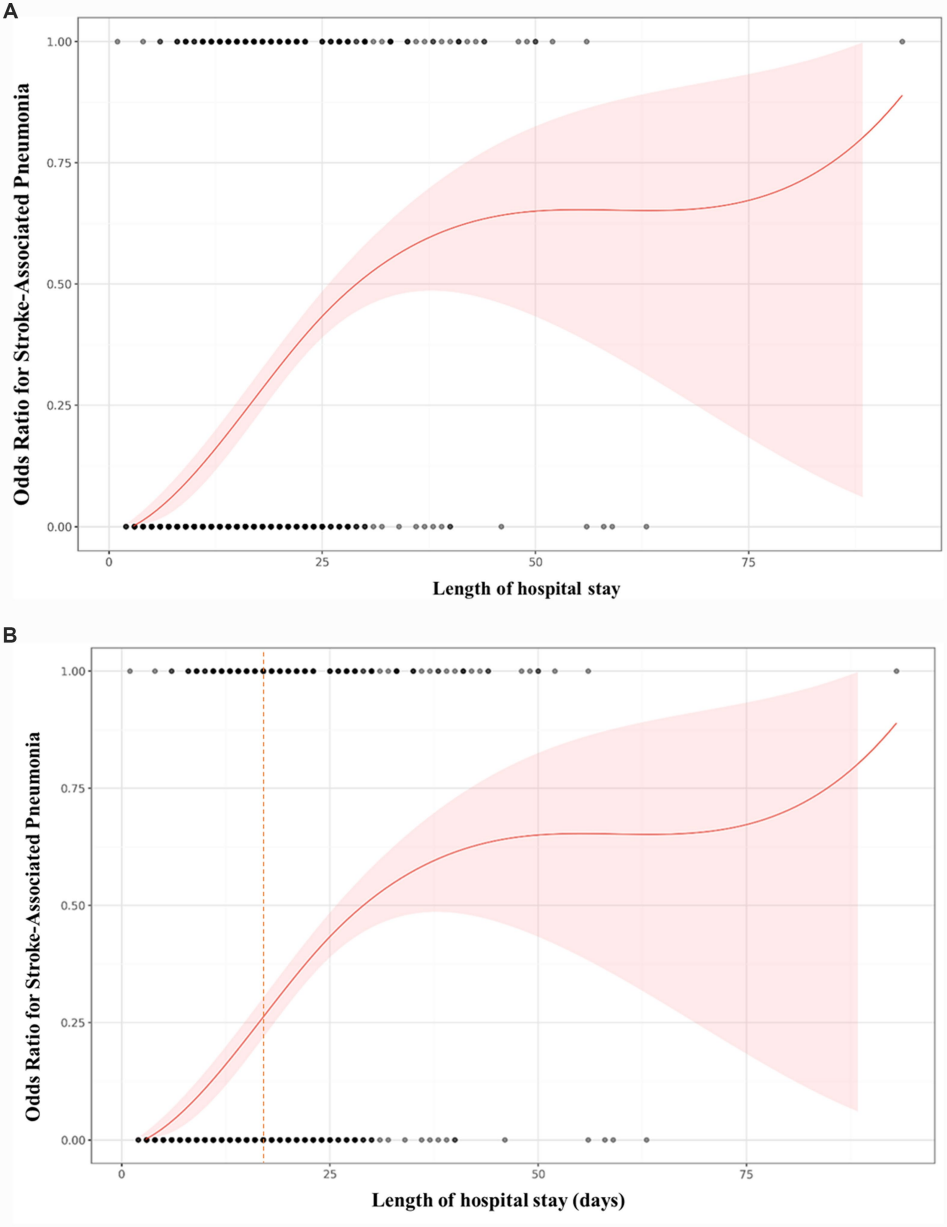
**(A, B)** Adjusted odds ratio (OR) for stroke-associated pneumonia (SAP) as a function of length of hospital stay (LOS, in days) from the two-piecewise logistic regression model (reference: LOS = 0 days). The solid red line shows the estimated OR, with the shaded area representing the 95% confidence interval. Black dots at the bottom indicate the distribution of observed LOS values. A vertical dashed line at 17 days marks the inflection point.

Figure 2 presents the nonlinear relationship between LOS and the OR for SAP. The x-axis denotes the length of hospital stay, while the y-axis indicates the odds ratio for the occurrence of SAP. The shaded area represents the 95% confidence interval. To evaluate potential nonlinearity in the association between LOS and SAP, a two-piecewise linear regression model was applied (Table 4). In the primary analysis using multiple imputation datasets, the inflection point was identified at 17 days. For LOS < 17 days, each additional day was associated with an 18.2% increase in SAP risk (*OR* = 1.182, 95% *CI*: 1.097–1.273, *p* < 0.0001); for LOS ≥ 17 days, the effect weakened, with only a 4.9% increase per day (*OR* = 1.049, 95% *CI*: 1.015–1.083, *p* = 0.0041). The log-likelihood ratio test yielded a *p* value of 0.010, indicating that the piecewise model fit the data better than the standard linear model, supporting a nonlinear relationship between LOS and SAP. Sensitivity analyses using the original dataset showed consistent trends, with an inflection point at 17.0 days (<17 days: *OR* = 1.181, 95% *CI*: 1.097–1.272, *p* < 0.0001; ≥17 days: *OR* = 1.015, 95% *CI*: 1.015–1.083, *p* = 0.0045; log-likelihood ratio test *p* = 0.010), further confirming the robustness of the main findings (Supplementary Table).

**Table 4:** Nonlinearity addressing between LOS and SAP.

Analysis model	OR, 95%CI, *p* value
Fitting model by standard logistic regression model	1.082 (1.056, 1.108) < 0.0001
Fitting model by two-piecewise linear model	
Inflection point of LOS	17.0
< 17.0	1.182 (1.098, 1.273) < 0.0001
≥ 17.0	1.049 (1.015, 1.084) 0.0040
P for log likely ratio test	0.010

Our covariate adjustment strategy is the same as Table 3.

Exploratory subgroup analyses revealed statistical evidence of effect modification by sex (*P* for interaction = 0.025), history of stroke/TIA (*P* for interaction = 0.001), coronary heart disease (*P* for interaction = 0.010), and OCSP classification (*P* for interaction = 0.005). The adjusted association between longer length of hospital stay (LOS) and increased risk of stroke-associated pneumonia (SAP) appeared stronger in females, patients without prior stroke/TIA history, patients without coronary heart disease, and those with posterior circulation infarct (POCI) subtype (Table 5).

**Table 5:** Exploratory subgroup analyses of the association between length of hospital stay (per 1-day increase) and stroke-associated pneumonia.

Stratification variable	Stratum	*n*	Adjusted OR (95% *CI*)	*p* value	P-interaction
Age (years)	<65	562	1.108 (1.077, 1.141)	<0.001	0.697
	>65	364	1.099 (1.066, 1.134)	<0.001	
Sex	Male	340	1.072 (1.039, 1.107)	<0.001	**0.025**
	Female	586	1.127 (1.095, 1.159)	<0.001	
Dysphagia	No	740	1.086 (1.057, 1.116)	<0.001	0.519
	Yes	186	1.106 (1.054, 1.159)	<0.001	
Hypertension	No	385	1.095 (1.062, 1.128)	<0.001	0.460
	Yes	541	1.112 (1.080, 1.146)	<0.001	
DM	No	650	1.093 (1.067, 1.120)	<0.001	0.168
	Yes	276	1.131 (1.083, 1.180)	<0.001	
History of stroke/TIA	No	664	1.085 (1.061, 1.110)	<0.001	**0.001**
	Yes	262	1.184 (1.123, 1.248)	<0.001	
CHD	No	840	1.115 (1.090, 1.141)	<0.001	**0.010**
	Yes	86	1.026 (0.969, 1.086)	0.374	
Smoking	Non-current	666	1.099 (1.074, 1.126)	<0.001	0.486
	Yes	260	1.119 (1.070, 1.171)	<0.001	
OCSP	LACI	191	1.093 (1.043, 1.144)	<0.001	**0.005**
	PACI	338	1.074 (1.042, 1.107)	<0.001	
	TACI	29	Not estimable (low events)	N/A	
	POCI	221	1.202 (1.130, 1.278)	<0.001	
NIHSS score	0–4	603	1.088 (1.051, 1.127)	<0.001	0.239
	5–12	227	1.092 (1.053, 1.132)	<0.001	
	>12	96	1.043 (1.000, 1.087)	0.051	

Adjusted odds ratios (ORs) and corresponding *p* values were derived from separate multivariable logistic regression models, each including the fully adjusted primary model (as described in Table 3) plus one single LOS × stratification variable interaction term. P for interaction values were obtained from likelihood ratio tests. Significant interactions (*p* < 0.05) are shown in bold. The TACI subgroup was not estimable due to small sample size and low event rate.

OR, odds ratio; CI, confidence interval; LOS, length of hospital stay; SAP, stroke-associated pneumonia; CHD, coronary heart disease; DM, diabetes mellitus; NIHSS, National Institutes of Health Stroke Scale; OCSP, Oxfordshire Community Stroke Project; TIA, transient ischemic attack.

Sensitivity analyses using multiple imputation for missing data (primarily OCSP variables, missing rate 15.87%) generated five imputed datasets, pooled via Rubin’s rules. The pooled adjusted OR was 1.083 (95% CI: 1.057–1.109, *p* < 0.001), confirming robust results. Variance inflation factors (VIF) were all <5, indicating no substantial multicollinearity (Supplementary Table S1).

## Discussion

This secondary analysis of prospectively collected cohort data involving a large sample of 926 patients examined the association between LOS and SAP. The study employed sophisticated multiple imputation methodology to handle missing data, ensuring robust and reliable analytical results. The findings confirmed a significant association between hospital length of stay and the risk of developing stroke-associated pneumonia. Further nonlinear analysis revealed the complexity of this relationship, showing a stronger association for shorter lengths of stay compared to longer ones. These results underscore the necessity for clinicians to implement pneumonia prevention strategies early in the hospitalization process.

Although no existing study has directly quantified the association between LOS as an exposure and SAP as an outcome, several real-world studies indirectly support the role of LOS as a proxy for healthcare quality and complication burden. For instance, Poll et al. (9) reported that the implementation of a specialized stroke unit in Brazil led to reduced hospitalization duration and lower in-hospital mortality, suggesting that streamlined care pathways may mitigate adverse events. Similarly, Labán-Seminario et al. (12) observed increasing length of stay trends in Peru correlated with higher in-hospital mortality, highlighting the need for efficient resource utilization. However, these studies did not adequately adjust for key confounders such as NIHSS score or dysphagia, potentially biasing effect estimates.

In contrast, our study adjusts for critical clinical variables including neurological severity and swallowing function, enhancing causal inference. Moreover, we uniquely demonstrated a nonlinear relationship, with accelerated SAP risk within the first several days—consistent with the clinical notion of an “early vulnerability window” during which impaired airway protection and stress-induced immunosuppression heighten infection susceptibility (13). Furthermore, our subgroup analysis reveals a clinically significant bidirectional interaction pattern: a history of prior stroke or TIA significantly enhances the positive association between length of stay and SAP, while coronary artery disease notably weakens this relationship. This contrast highlights the differential modifying effects of various underlying conditions on the risk trajectory of post-stroke infections. For patients with a prior history of stroke or TIA, diminished cerebral reserve, covert swallowing difficulties, and delayed neurological recovery may lead to an increased accumulation of risks such as aspiration and bed-related complications during hospitalization (14), thereby amplifying the “dose–response” effect of length of stay on SAP. Conversely, patients with coronary artery disease, often classified as high-risk cardiovascular individuals, typically receive more rigorous multi-system monitoring and early interventions (such as swallowing assessments, bed elevation, oral care, and early rehabilitation) (15), along with the pulmonary protective effects of routinely used statins and renin-angiotensin system inhibitors, which collectively mitigate the infection risk during prolonged hospitalization (16). Notably, the coronary artery disease group had a smaller sample size and may be subject to survival bias, which could partly explain the attenuation of effects. These findings suggest that length of stay should not be viewed merely as a temporal variable but rather understood as a “risk exposure window,” the clinical significance of which is highly dependent on patients’ baseline vulnerability and the intensity of preventive interventions they receive.

This study has several methodological strengths. First, its prospective cohort design ensured standardized data collection and minimized recall and selection biases. Second, comprehensive adjustment for potential confounders—including age, sex, comorbidities, NIHSS score, dysphagia, and OCSP subtype—improved estimate accuracy, with variance inflation factors (VIF < 5) confirming absence of multicollinearity. Third, multiple imputation was used to handle missing data, and consistent results across original and imputed datasets (see Supplementary Tables S2, S3) enhanced the robustness of conclusions. Fourth, to our knowledge, this is the first study to directly examine the nonlinear association between LOS as the primary exposure and SAP risk using a threshold effect model, though prior studies have indirectly suggested steeper risks with prolonged hospitalization. Finally, the study population from a major tertiary hospital in China, with a large sample size, offers good representativeness and generalizability.

Several limitations should be acknowledged. First, as a secondary analysis of registry data, certain established predictors of stroke-associated pneumonia—such as chronic obstructive pulmonary disease, congestive heart failure, pre-stroke functional status (modified Rankin Scale), and Glasgow Coma Scale—were not recorded and could not be adjusted for. Although we controlled for key available factors, including atrial fibrillation, NIHSS score (a robust proxy for stroke severity and consciousness), dysphagia, age, and sex, residual confounding by these unmeasured variables remains possible. Second, the exact onset date of SAP during hospitalization was unavailable, preventing precise temporal analysis and full disentanglement of bidirectional causality (i.e., whether prolonged LOS increases SAP risk or SAP extends LOS). Despite multivariable adjustment, reverse causation may partly influence our findings. Third, LOS is a composite measure affected not only by clinical severity but also by non-clinical factors (e.g., insurance policies, bed availability, family support), raising concerns about its role as an independent exposure rather than a mediator. Additionally, SAP diagnosis relied on clinical and imaging criteria without standardized microbiological confirmation, potentially introducing misclassification bias. Fourth, the single-center design and data collection ending in 2012 limit generalizability to contemporary practice, where increased use of reperfusion therapies may have altered LOS distribution and its association with SAP. Finally, categorization of LOS in supplementary analyses was based on data distribution and the identified inflection point rather than more flexible methods (e.g., restricted cubic splines), though primary nonlinear findings relied on continuous and two-piecewise models for robustness.

The data were collected between 2009 and 2012, prior to widespread adoption of endovascular thrombectomy and modern stroke unit protocols in many Chinese centers. While this limits generalizability to contemporary high-resource settings, the observed association between prolonged LOS and SAP risk likely remains relevant in healthcare systems where extended hospitalization persists due to resource or reimbursement constraints. Additionally, several exploratory subgroups [e.g., coronary heart disease (*n* = 86) and TACI subtype (*n* = 29)] were small, leading to wide confidence intervals and reduced precision. These stratified estimates should be considered hypothesis-generating.

Our findings carry important implications for clinical practice and healthcare management. LOS is not merely a measure of efficiency but also a strong predictor of SAP, particularly during the initial 17 days when risk rises most rapidly. This suggests that hospitals should intensify respiratory care, swallowing assessment, and infection prevention immediately upon admission—such as structured bedside dysphagia screening, head-of-bed elevation, oral care, and early enteral nutrition. Furthermore, recognizing the nonlinear risk pattern can guide resource allocation: patients expected to stay beyond 17 days may transition earlier to rehabilitation-focused care to minimize unnecessary antibiotic use and invasive procedures. Policymakers should avoid incentivizing shorter LOS at the expense of infection control quality, as cautioned by Sarraj et al. (17), who emphasized the need to balance aggressive interventions like thrombectomy with post-procedural complication management. Future research could integrate biomarkers (e.g., SIRI, IL-6) into dynamic prediction models for personalized SAP risk stratification and intervention.

## Conclusion

In this secondary analysis of a historical single-center registry, length of hospital stay was positively associated with stroke-associated pneumonia risk in a nonlinear manner, with a steeper increase during the first 17 days of hospitalization. These findings, while limited by the older cohort and potential residual confounding, suggest value in intensified respiratory monitoring and infection prevention early during admission, particularly in settings with prolonged hospitalization. Future studies in contemporary cohorts are needed to confirm this pattern and guide optimized hospitalization strategies.

## Data Availability

The original contributions presented in the study are included in the article/Supplementary material, further inquiries can be directed to the corresponding author.
