# Supplementary material for: Analyzing the nonlinear association between length of hospital stay and post-stroke pneumonia risk a secondary analysis of the Henan Province stroke registry
Source: Front Neurol. 2026 Feb 4;17:1711762. doi: 10.3389/fneur.2026.1711762 (PMC12913055; doi:10.3389/fneur.2026.1711762)
Supplement: Supplementary file 1 [file Table_1.docx]

**Statistical Analysis Supplemental Table**

**Table S1. Missing Data Analysis**

| **Variable** | **Complete (n)** | **Missing (n)** | **Missing (%)** | **Handling Method** |
| --- | --- | --- | --- | --- |
| OCSP | 779 | 147 | 15.87 | missForest imputation |
| AGE.CS | 926 | 0 | 0.00 | missForest imputation |
| SEX | 926 | 0 | 0.00 | missForest imputation |
| DYSPHAGIA | 926 | 0 | 0.00 | missForest imputation |
| AF | 926 | 0 | 0.00 | missForest imputation |
| HYPERTESION | 926 | 0 | 0.00 | missForest imputation |
| DM | 926 | 0 | 0.00 | missForest imputation |
| DYSLIPIDEMIA | 926 | 0 | 0.00 | missForest imputation |
| CVDS | 926 | 0 | 0.00 | missForest imputation |
| CHD | 926 | 0 | 0.00 | missForest imputation |
| SMOKING | 926 | 0 | 0.00 | missForest imputation |
| NIHSS.CS1 | 926 | 0 | 0.00 | missForest imputation |

*Table S1. Analysis of missing data for study variables. Variables with missing rate ≥40% were excluded. When any variable has missing rate ≥5%, all covariates are imputed using missForest algorithm regardless of their individual missing rates. If all variables have missing rate <5%, only direct deletion of missing values is used. Final sample size after processing: 926.*
