# Supplementary material for: Analyzing the nonlinear association between length of hospital stay and post-stroke pneumonia risk a secondary analysis of the Henan Province stroke registry
Source: Front Neurol. 2026 Feb 4;17:1711762. doi: 10.3389/fneur.2026.1711762 (PMC12913055; doi:10.3389/fneur.2026.1711762)
Supplement: Supplementary file 2 [file Table_2.docx]

**Statistical Analysis Supplemental Tables**

***Supplemental Table S2. Comparison of Original and Imputed Data***

| **Variable** | **Original Data** | **Imputation 1** | **Imputation 2** | **Imputation 3** | **Imputation 4** | **Imputation 5** | **Test** | **P-value** |
| --- | --- | --- | --- | --- | --- | --- | --- | --- |
| CVDS |  | | | | | | N/A (No missing values) | **-** |
| *0.0* | 664 (71.7%) | 664 (71.7%) | 664 (71.7%) | 664 (71.7%) | 664 (71.7%) | 664 (71.7%) |  |  |
| *1.0* | 262 (28.3%) | 262 (28.3%) | 262 (28.3%) | 262 (28.3%) | 262 (28.3%) | 262 (28.3%) |  |  |
| DYSLIPIDEMIA |  | | | | | | N/A (No missing values) | **-** |
| *0.0* | 926 (100.0%) | 926 (100.0%) | 926 (100.0%) | 926 (100.0%) | 926 (100.0%) | 926 (100.0%) |  |  |
| CHD |  | | | | | | N/A (No missing values) | **-** |
| *0.0* | 840 (90.7%) | 840 (90.7%) | 840 (90.7%) | 840 (90.7%) | 840 (90.7%) | 840 (90.7%) |  |  |
| *1.0* | 86 (9.3%) | 86 (9.3%) | 86 (9.3%) | 86 (9.3%) | 86 (9.3%) | 86 (9.3%) |  |  |
| SMOKING |  | | | | | | N/A (No missing values) | **-** |
| *0.0* | 666 (71.9%) | 666 (71.9%) | 666 (71.9%) | 666 (71.9%) | 666 (71.9%) | 666 (71.9%) |  |  |
| *1.0* | 260 (28.1%) | 260 (28.1%) | 260 (28.1%) | 260 (28.1%) | 260 (28.1%) | 260 (28.1%) |  |  |
| OCSP |  | | | | | | Chi-square | **0.423** |
| *0.0* | 191 (24.5%) | 195 (21.1%) | 196 (21.2%) | 202 (21.8%) | 206 (22.2%) | 197 (21.3%) |  |  |
| *1.0* | 338 (43.4%) | 473 (51.1%) | 479 (51.7%) | 467 (50.4%) | 450 (48.6%) | 459 (49.6%) |  |  |
| *2.0* | 29 (3.7%) | 30 (3.2%) | 29 (3.1%) | 29 (3.1%) | 30 (3.2%) | 30 (3.2%) |  |  |
| *3.0* | 221 (28.4%) | 228 (24.6%) | 222 (24.0%) | 228 (24.6%) | 240 (25.9%) | 240 (25.9%) |  |  |
| AGE.CS |  | | | | | | N/A (No missing values) | **-** |
| *0.0* | 562 (60.7%) | 562 (60.7%) | 562 (60.7%) | 562 (60.7%) | 562 (60.7%) | 562 (60.7%) |  |  |
| *1.0* | 364 (39.3%) | 364 (39.3%) | 364 (39.3%) | 364 (39.3%) | 364 (39.3%) | 364 (39.3%) |  |  |
| NIHSS.CS1 |  | | | | | | N/A (No missing values) | **-** |
| *0.0* | 603 (65.1%) | 603 (65.1%) | 603 (65.1%) | 603 (65.1%) | 603 (65.1%) | 603 (65.1%) |  |  |
| *1.0* | 227 (24.5%) | 227 (24.5%) | 227 (24.5%) | 227 (24.5%) | 227 (24.5%) | 227 (24.5%) |  |  |
| *2.0* | 96 (10.4%) | 96 (10.4%) | 96 (10.4%) | 96 (10.4%) | 96 (10.4%) | 96 (10.4%) |  |  |
| SEX |  | | | | | | N/A (No missing values) | **-** |
| *0.0* | 340 (36.7%) | 340 (36.7%) | 340 (36.7%) | 340 (36.7%) | 340 (36.7%) | 340 (36.7%) |  |  |
| *1.0* | 586 (63.3%) | 586 (63.3%) | 586 (63.3%) | 586 (63.3%) | 586 (63.3%) | 586 (63.3%) |  |  |
| DYSPHAGIA |  | | | | | | N/A (No missing values) | **-** |
| *0.0* | 740 (79.9%) | 740 (79.9%) | 740 (79.9%) | 740 (79.9%) | 740 (79.9%) | 740 (79.9%) |  |  |
| *1.0* | 186 (20.1%) | 186 (20.1%) | 186 (20.1%) | 186 (20.1%) | 186 (20.1%) | 186 (20.1%) |  |  |
| AF |  | | | | | | N/A (No missing values) | **-** |
| *0.0* | 926 (100.0%) | 926 (100.0%) | 926 (100.0%) | 926 (100.0%) | 926 (100.0%) | 926 (100.0%) |  |  |
| HYPERTESION |  | | | | | | N/A (No missing values) | **-** |
| *0.0* | 385 (41.6%) | 385 (41.6%) | 385 (41.6%) | 385 (41.6%) | 385 (41.6%) | 385 (41.6%) |  |  |
| *1.0* | 541 (58.4%) | 541 (58.4%) | 541 (58.4%) | 541 (58.4%) | 541 (58.4%) | 541 (58.4%) |  |  |
| DM |  | | | | | | N/A (No missing values) | **-** |
| *0.0* | 650 (70.2%) | 650 (70.2%) | 650 (70.2%) | 650 (70.2%) | 650 (70.2%) | 650 (70.2%) |  |  |
| *1.0* | 276 (29.8%) | 276 (29.8%) | 276 (29.8%) | 276 (29.8%) | 276 (29.8%) | 276 (29.8%) |  |  |

*Supplemental Table 2. Comparison between original data and imputed values across 5 imputation sets. For continuous variables, values are presented as mean ± standard deviation. Statistical comparisons were performed using one-way ANOVA for normally distributed variables (when 2×SD ≤ mean) or Kruskal-Wallis test for non-normally distributed variables. For categorical variables, values are presented as counts (percentage), and comparisons were performed using Chi-square test. Variables with missing values are highlighted. When imputation is needed, all covariates are imputed regardless of their missing percentage.*
