# Supplementary material for: Analyzing the nonlinear association between length of hospital stay and post-stroke pneumonia risk a secondary analysis of the Henan Province stroke registry
Source: Front Neurol. 2026 Feb 4;17:1711762. doi: 10.3389/fneur.2026.1711762 (PMC12913055; doi:10.3389/fneur.2026.1711762)
Supplement: Supplementary file 3 [file Table_3.docx]

**Statistical Analysis Supplemental Tables**

**Supplemental Table S3. Results from Individual Imputation Sets**

| **Analysis** | **Imputation 1** | **Imputation 2** | **Imputation 3** | **Imputation 4** | **Imputation 5** | **Pooled Result** |
| --- | --- | --- | --- | --- | --- | --- |
| X17 vs X18 (Unadjusted) | 1.104 (1.081, 1.128) P=<0.001 | 1.104 (1.081, 1.128) P=<0.001 | 1.104 (1.081, 1.128) P=<0.001 | 1.104 (1.081, 1.128) P=<0.001 | 1.104 (1.081, 1.128) P=<0.001 | 1.104 (1.081, 1.128) P=<0.001 |
| X17 vs X18 (Adjusted) | 1.084 (1.058, 1.110) P=<0.001 | 1.083 (1.057, 1.110) P=<0.001 | 1.083 (1.057, 1.109) P=<0.001 | 1.084 (1.058, 1.111) P=<0.001 | 1.083 (1.058, 1.110) P=<0.001 | 1.083 (1.057, 1.110) P=<0.001 |

*Supplemental Table 3. Results from each individual imputation dataset and pooled analysis using Rubin's rules. Effect size (95% CI) and P-value are shown for each imputation set (1-5) and the final pooled result.*
